# Reference Interval for Pulse Oxygen Saturation in Neonates at Different Altitudes: A Systematic Review

**DOI:** 10.3389/fped.2021.771750

**Published:** 2021-11-01

**Authors:** Bo Wang, Jia Zhang, Ya-Zhen Wu, Zhi-Hui Lu, Na Wang, Zhang-Bin Yu

**Affiliations:** ^1^Department of Pediatrics, The Affiliated Suqian First People's Hospital of Nanjing Medical University, Suqian, China; ^2^Department of Neonatology, Qinghai University Affiliated Hospital, Xining, China; ^3^Department of Obstetrics and Gynecology, Qinghai University Affiliated Hospital, Xining, China; ^4^Department of Neonatology, The Affiliated Obstetrics and Gynecology Hospital of Nanjing Medical University, Nanjing, China

**Keywords:** altitude, pulse oxygen saturation, SpO_2_, reference interval, predictive equations, systematic review, neonates

## Abstract

**Introduction:** The reference interval for pulse oxygen saturation (SpO_2_) in neonates born at high altitudes has not been defined to date. The purpose of this study was to systematically review published studies and determine the reference interval of SpO_2_ in neonates at different altitudes.

**Methods:** Databases of PubMed, Embase, Cochrane Library, Clinicaltrials.Gov, Chinese National Knowledge Infrastructure Database, Wanfang Database, Chinese Science Technology Journals Database, and Chinese Clinical Trial Registry were searched for studies reporting SpO_2_ in healthy neonates at different altitudes. Retrieval time was from inception of the database to August 16, 2021. The Agency for Healthcare Research and Quality checklist was used to evaluate the quality of studies. Python v3.8 was used to analyze the data. This systematic review was drafted in accordance with the guidelines of the Preferred Reporting Items for Systematic Reviews and Meta-Analyses (PRISMA) statement.

**Results:** Seven cross-sectional studies, published between 1991 and 2020, were identified. They were from US, Mexico, Israel, Ecuador, and China. Three studies were rated as high quality and four as moderate quality. The mean SpO_2_ (with standard deviation or standard error) of neonates born in 40 different altitudes (ranging from 25 meters to 3,100 meters) were obtained. The prediction equation for calculation of the lower limit of the reference interval was established, and the reference intervals for SpO_2_ at different altitudes were determined.

**Conclusions:** In healthy neonates, the lower limit of the reference interval of SpO_2_ decreases with increase in altitude. High-quality prospective studies are need to confirm our findings.

## Introduction

Knowledge of the reference interval of pulse oxygen saturation (SpO_2_) in neonates is crucial for accurate identification of neonatal hypoxemia (1). At sea level and adjacent altitudes, 2.5% of neonates have SpO_2_ below 95%, and so this value is used as the cutoff for identifying hypoxemia at these altitudes (2). Establishment of such reference values is important for standardized clinical management (3). However, at present, there is no recommended reference interval for SpO_2_ in high altitude areas. The standard recommended for the plains is not applicable because atmospheric pressure and partial pressure of oxygen decrease as altitude increases and will therefore cause decrease in SpO_2_ (4). Guo et al. (5) suggested that the value corresponding to the 2.5th percentile of the SpO_2_ distribution range in healthy neonates born at different altitudes be used as the cutoff value. Unfortunately, there are not many studies on the SpO_2_ distribution range in healthy neonates born at different altitudes, especially during the period from 24 h after birth to discharge, which is defined as the stable period after birth. No systematic reviews of published studies have been performed.

The purpose of this study was to review the published data and establish the reference interval for SpO_2_ in the stable period of healthy neonates at different altitudes. The findings of this study will allow evidence-based medical decision making in high altitude areas.

## Methods

This systematic review of all studies on SpO_2_ in healthy neonates at different altitudes during the stable period was reported according to Preferred Reporting Items for Systematic Reviews and Meta-Analyses (PRISMA) guidelines (6).

### Data Sources and Literature Search

To identify articles on SpO_2_ in neonates in the stable period after birth, we performed a literature search of the databases of PubMed, Embase, Cochrane Library, Clinicaltrials.Gov, Chinese National Knowledge Infrastructure Database (CNKI), Wanfang Database, Chinese Science Technology Journals Database (VIP), and Chinese Clinical Trial Registry (ChiCTR). Papers in any language, published from inception of the database to August 16, 2021, were eligible for consideration. The search terms were “altitude,” “neonate,” and “oximetry,” in combination with Medical Subject Headings (MeSH) terms and other equivalent terms; logical symbols, wildcards, and range operators were used to compile search formulas. The search strategy used in PubMed is described in online Supplementary Material 1.

### Inclusion and Exclusion Criteria

Articles were eligible for inclusion in this review if (1) the study participants were healthy asymptomatic neonates born at a specified altitude; (2) a pulseoximetry (POX) was used to measure SpO_2_ on the right hand (pre-ductal) and/or either foot (post-ductal); (3) measurement was made during the stable period, i.e., 24 h after birth to before discharge; and (4) measurement was made while the neonate was inhaling room air (i.e., no oxygen supplementation). Studies were excluded if (1) they did not mention the measurement site (pre-ductal or post-ductal); (2) measurement was made within 24 h of birth; (3) data could not be extracted; (4) gestational age was <34 weeks; or (5) altitude was not specified.

### Data Collection and Extraction

Two researchers independently conducted the literature search and extracted and cross-checked the data. Disagreements were settled by discussion and, if necessary, by consultation with the third researcher. Duplicate articles were first eliminated. Then, the researchers read the titles and abstracts of all articles and discarded obviously irrelevant papers. Finally, the full text of the remaining papers were read, and studies that satisfied all eligibility criteria were selected.

The following data were extracted from the selected articles: (1) basic information regarding the study, i.e., name of first author, publication date, study period, study location and altitude, sample size; (2) baseline characteristics of the study participants and the methods used, i.e., gestational age, POX type, measurement site (hand, foot), time of measurement, resting state at the time of measurement, and summary statistics. If multiple SpO_2_ measurements had been made during the stable period, only the values recorded closest to the time of birth was extracted. Similarly, if measurements had been made during different neonatal resting states (e.g., awake but quiet, light sleep, deep sleep, and so on), only the values recorded in the awake but quiet state was extracted.

### Quality Assessment

Study quality was assessed using the checklist of the Agency for Healthcare Research and Quality (AHRQ) (7). This checklist comprises 11 questions that assess different aspects of the study such as definition of information sources, inclusion and exclusion criteria, the time period and continuity of identifying patients, blinding of personnel, quality assurance, confounding and missing data, patient response rates and completeness of response. The response to each question may be “yes,” “no,” or “unclear.” A score of 1 point is assigned for “yes” and 0 points for “no” or “unclear.” Thus, the total score can range from 0 to 11, with 8–11 indicating high quality, 4–7 indicating moderate quality, and 0–3 indicating low quality. In case of discordance in scoring, the third researcher's decision was final.

### Statistical Analysis

The mean ± standard deviation (SD) of SpO_2_, or the 2.5th percentile value of the SpO_2_ distribution range, were recorded. If the study results were expressed as the mean value ± standard error (SE), the SD was derived using the formula: $SD\;=\;SE\;\times \;\sqrt{n}$ (where n is the sample size). If the data showed a skewed distribution, the lower limit of the reference interval was the value corresponding to the 2.5th percentile in the distribution range of SpO_2_ of healthy neonates born at different altitudes. If the data were normally distributed, the lower limit value corresponds to the −2SD value (mean value-2^*^SD). Therefore, the reference interval of SpO_2_ was defined as: the lower limit value < SpO_2_ ≤ 100%.

All data were entered into Microsoft Excel. Python v3.8 was used to read the data in Microsoft Excel for analysis. Then, the curve-fitting function in the numerical calculation library was used to perform polynomial fitting on the lower limit values. Next, the altitude data were taken as the parameters of the fitted function, and the predictive equations were obtained for the lower limit values.

## Results

### Study Selection

**Figure 1:**
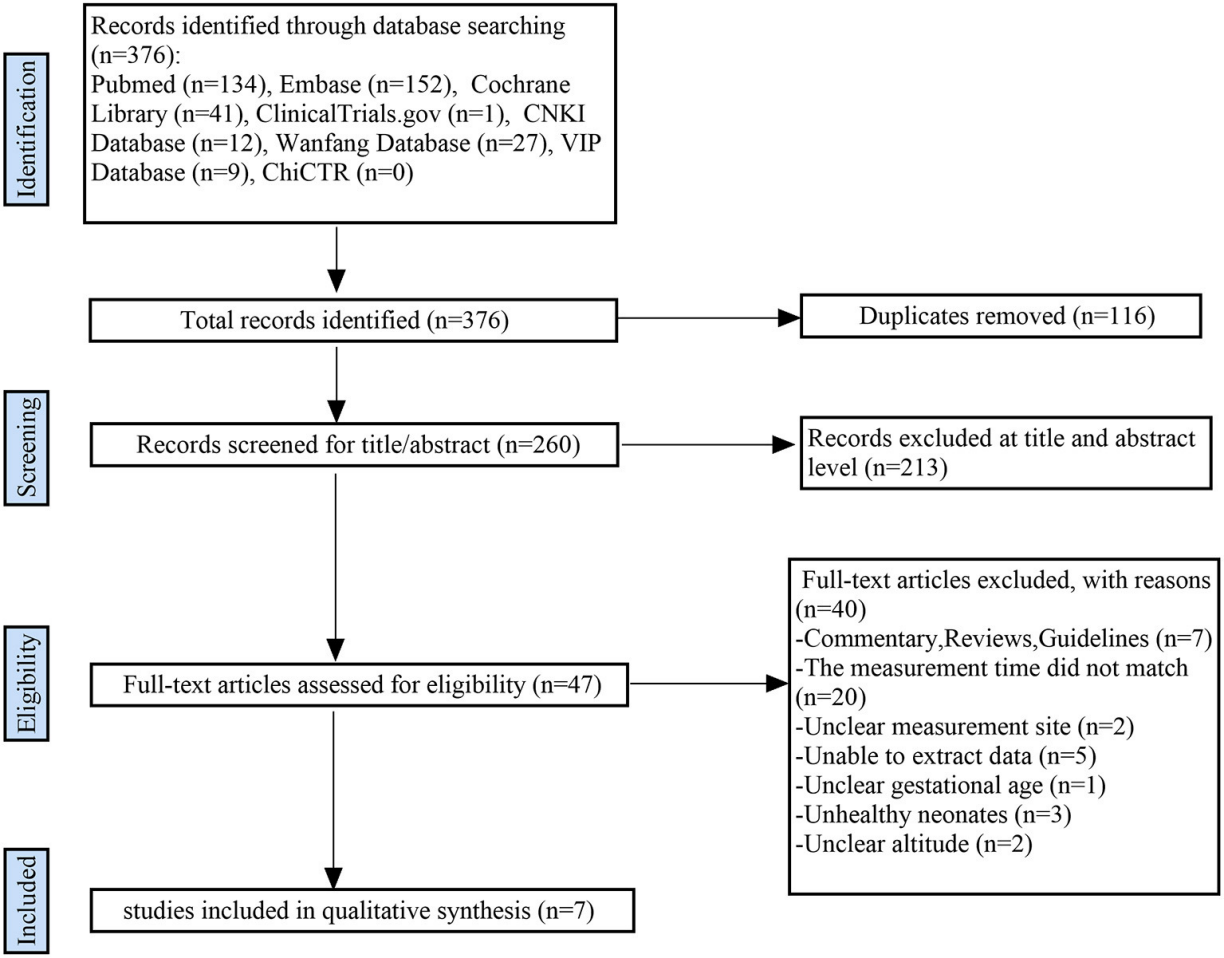
Flow diagram showing study selection process.

A total of 376 articles were initially retrieved. From these, 116 duplicate articles and 213 obviously irrelevant articles were first screened out. Of the remaining 47 articles, seven satisfied all eligibility criteria and were included in this systematic review (5, 8–13). Figure 1 shows the literature screening process.

### Study Characteristics

All seven articles were cross-sectional studies: three were from the US, one from Mexico, one from Israel, one from Ecuador, and one from China. One study was in Spanish and the rest were in English. The sample sizes ranged from 15 to 41,097, and the altitudes ranged from 25 to 3,100 meters above sea level. Four studies did not specify the study period. Three studies did not specify the neonatal resting state during measurement. Table 1 summarizes the characteristics of the seven studies.

**Table 1:** Basic characteristics of included studies.

**Reference**	**Year^**a**^**	**Study time**	**Country**	**Altitude (m)**	**Sample size**	**Gestational age**	**POX type**	**Measurement site**	**Measurement time**	**Summary statistics**	**State during measurement**
Thilo et al. (8)	1991	Not mentioned	USA	1,610	150	Term infants	Ohmeda Biox 3700	Post ductal	24–48 h after birth	Mean, SE	Awake but quiet
Niermeyer et al. (9)	1993	Not mentioned	USA	3,100	15	Term infants	Ohmeda Biox 3700	Post ductal	24–48 h after birth	Mean, SD	Awake but quiet
TapiaRombo et al. (10)	2008	January to April 2004	Mexico	2,240	89	Term infants	Internal Electrical CE 0123	Post ductal	24 h to 7 d after birth	Mean, SD	Not mentioned
Han et al. (11)	2013	March to December 2012	USA	806	1,062	Term infants	Masimo SET Radical	Pre ductal and post ductal	24 h after birth to before discharge	Mean, SD	Not mentioned
Samuel et al. (12)	2013	Not mentioned	Israel	25 and 780	199	Term infants	Masimo Radical-7	Pre ductal and post ductal	24–72 h after birth	Mean, SD	Sleep or awake but quiet
González-Andrade et al. (13)	2018	4 months, but month/year not specified	Ecuador	2,820	963	Term infants	EDAN M3	Pre ductal and post ductal	24–48 h after birth	Mean, SD	Awake but quiet
Guo et al. (5)	2020	August 2015 to June 2016	China	267 to 2,202	41,097	≥35 weeks	Masimo RAD5	Pre ductal and post ductal	24 h after birth	Mean, SD	Not mentioned

a *Year of publication*.

*POX, pulseoximetry; SD, standard deviation; SE, standard error*.

### Quality Assessment

By the AHRQ scores, three studies were graded as high quality and four as moderate quality (Table 2).

**Table 2:** Quality assessment of studies by the Agency for Healthcare Research and Quality (AHRQ) scoring system.

**Reference**	**A**	**B**	**C**	**D**	**E**	**F**	**G**	**H**	**I**	**J**	**K**	**Total score**
Thilo et al. (8)	1	1	0	0	1	0	1	0	1	1	1	7
Niermeyer et al. (9)	1	1	0	0	1	0	1	0	1	1	1	7
Tapia-Rombo et al. (10)	1	1	1	0	1	0	1	0	0	1	0	6
Han et al. (11)	1	1	1	1	1	0	1	0	1	1	1	9
Samuel et al. (12)	1	1	0	1	1	0	1	1	1	1	1	9
González-Andrade et al. (13)	1	1	0	0	1	0	1	0	0	1	0	5
Guo et al. (5)	1	1	1	1	1	1	1	0	0	1	0	8

*A: Does the study clearly define of the source of information (survey, record review)? B: Does the study list the inclusion and exclusion criteria for exposed and unexposed subjects (cases and controls) or reference to previous publications? C: Is the time period for identifying patients defined? D: Were the participants consecutively enrolled or was the study population-based? E: Were the evaluators of subjective components blinded to other aspects of the status of the participants? F: Were assessments undertaken for quality assurance purposes (e.g., test/retest of primary outcome measurements) described? G: Were patient exclusions from analysis, if any, explained? H: Does the study describe how confounding was assessed and/or controlled? I: Does the study explain how missing data were handled in the analysis? J: Were patient response rates and completeness of data collection summarized. K: Does the study clarify what follow-up, if any, was expected and the percentage of patients for which incomplete data or follow-up was obtained?*

### Data Extraction

The mean (with SD or SE) SpO_2_ of healthy neonates born at 40 different altitudes were collected from the seven studies. Of these, three studies had only post-ductal data. Table 3 presents all SpO_2_ data expressed as means ± SD.

**Table 3:** SpO_2_ in healthy neonates during the stable period at different altitudes.

**Altitude (m)**	**Sample size**	**Pre-ductal mean (%)**	**Pre-ductal SD**	**Post-ductal mean (%)**	**Post-ductal SD**
25	119	98.28	1.14	98.9	1.16
267	832	98.3	1.2	98.6	1.2
383	868	97.3	1.4	97.6	1.4
408	1,171	98.1	1.5	98.3	1.5
553	406	96.7	1.5	96.5	1.6
640	293	97.2	1.4	96.8	1.4
730	462	96.8	1.8	97.4	1.7
780	80	97.86	1.58	98.49	1.35
806	1,062	98.5	1.33	98.6	1.32
907	856	97	1.8	97.6	1.9
1,077	1,587	97.4	1.6	97.8	1.6
1,135	1,003	96.1	2	96.4	1.9
1,217	1,969	96.8	1.6	97	1.5
1,308	1,822	94.9	1.5	95.1	1.5
1,323	1,925	95.8	1.4	96.1	1.4
1,356	623	97.1	1.5	97.5	1.6
1,401	863	96	1.8	96.1	1.7
1,417	1,452	96.6	1.9	96.9	1.9
1,427	2,371	96.3	1.6	96.7	1.6
1,457	805	96.9	1.8	96.6	1.8
1,547	1,890	96	1.8	96.2	1.7
1,565	850	96.2	1.7	96.4	1.7
1,588	445	97.1	1.9	97.4	1.9
1,610	150	ND	ND	92.7	2.4
1,647	1,165	95.6	2.2	95.7	2.1
1,665	381	97.4	2	97.1	2
1,667	1,054	96.3	2	96.4	2
1,670	619	96.4	2.1	96.7	2
1,673	2,044	95.2	2.1	95.4	2
1,683	6,625	94.2	1.9	94.9	1.8
1,733	1,167	95.6	1.8	95.3	1.8
1,734	3,255	96.3	1.8	96.5	1.8
2,005	455	96.1	2.1	96.7	2.1
2,060	681	95.9	2	96.3	2.1
2,199	481	96.9	1.9	96.1	2.1
2,201	289	93.3	2.4	95.7	2.3
2,202	388	96.4	2.3	96.7	2.2
2,240	89	ND	ND	93.5	2
2,820	963	92.77	3.03	93.76	2.83
3,100	15	ND	ND	88.8	1.8

*ND, No data available*.

### Data Analysis

Because the altitude varied between the studies, meta-analysis could not be performed. The pre-ductal and post-ductal data were analyzed separately. The altitude, mean (±SD) SpO_2_, and sample size were entered into Microsoft Excel, and the −2SD value of SpO_2_ for each altitude was calculated. The “xlrd” module of Python v3.8 was used to read the altitude column data, mean column data, and −2SD column data in the Microsoft Excel file; this was stored in the “list” collection. Then the curve-fitting function “polyfit” in the “numpy” numerical calculation library was used to perform polynomial fitting based on the least squares method on the −2SD column data. The “polyfit” function has three required parameters, namely abscissa data, ordinate data, and polynomial order, and its return value is the fitting function. After comparison, a third-order polynomial was selected as the fitting function. Then the “list” set of altitude data was used as the parameter of the fitting function, and the prediction equations for the lower limits for pre-ductal and post-ductal SpO_2_ were obtained:

For pre-ductal SpO_2_:


$$\begin{array}{l} \text{Sp}{\text{O}}_{2}\left(\text{\%}\right)\;=7.8\;\times \;10^{-10}\times {\left(\text{H}\right)}^{3}\;+\;2.75\times 10^{-6}\;\times \;{\left(\text{H}\right)}^{2}\\ \;-\;0.00486\;\times \;\left(\text{H}\right)\;+\;96.45 \end{array}$$

For post-ductal SpO_2_:


$$\begin{array}{l} \text{Sp}{\text{O}}_{2}\left(\text{\%}\right)\;=6.612\;\times \;10^{-10}\;\times \;{\left(\text{H}\right)}^{3}\;+\;2.482\;\times \;10^{-6}\\ \;\times {\left(\text{H}\right)}^{2}\;-\;0.004955\;\times \;\left(\text{H}\right)\;+\;96.86, \end{array}$$

where H stands for altitude, in meters.

After the lower limits for pre-ductal and post-ductal SpO_2_ at each altitude was calculated, the smallest integer of the two values was selected as the final lower limit of SpO_2_ at each altitude. For example, at altitude 2,500 meters, the lower limit values of pre-ductal and post-ductal SpO_2_ were 89.3 and 89.25%, respectively, and so the reference interval was: 89% < SpO_2_ ≤ 100%.

**Figure 2:**
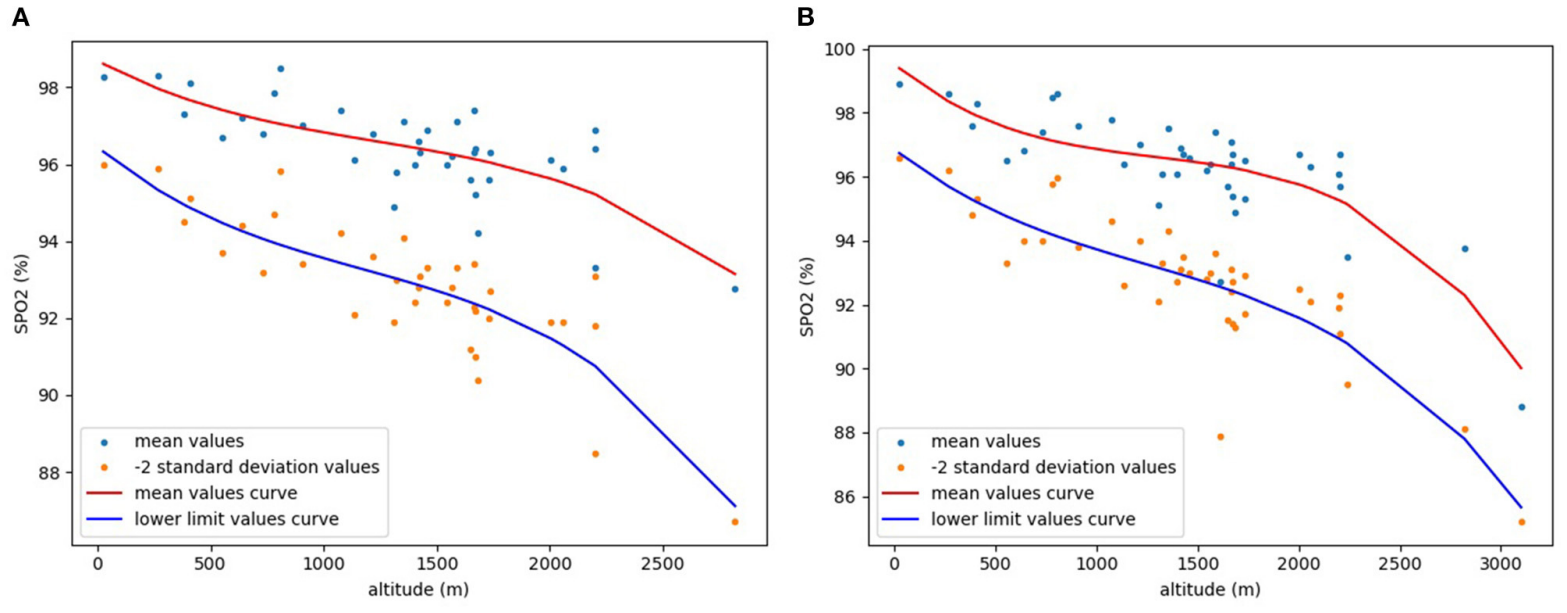
Relationship between altitude and mean and lower limit values of SpO_2_ in healthy neonates. **(A)** pre-ductal; **(B)** post-ductal.

Finally, the “pyplot” function in the visualization library “Matplotlib” was used to draw a −2SD scatter plot and a fitting curve of the lower limit values. For comparison, a scatter plot of the mean values and a fitting curve of the mean values was also drawn using the same method (Figure 2). Mean values scatter plot in Figure 2 is drawn from collected data, and mean values curve is obtained by curve fitting the collected mean values. The scatter plot of −2SD values in Figure 2 is calculated and drawn from the collected data, and lower limit values curve is obtained by curve fitting the −2SD values. As is clear from Figure 2, both pre-ductal and post-ductal SpO_2_ show a clear downward trend with increasing altitude.

## Discussion

This study aimed to determine the reference intervals for neonatal SpO_2_ at different altitudes. The results showed that the lower limit of the reference interval decreases as altitude increases. It is clear that the reference interval used in the plains is inappropriate for higher altitudes.

Physiologically, the most significant changes in the neonatal period are in the path of intracardiac blood flow and pulmonary circulation. Immediately after birth, the lungs change from being fluid filled to air filled. Pulmonary blood flow increases, and the foramen ovale and ductus arteriosus close, preventing shunting of blood. The respiratory system must respond to a rapid increase in metabolic rate and a gradual change in partial pressure of oxygen from fetal to postnatal period, while continuing the final steps of structural maturation of the alveolar gas exchange unit. Oxygen plays a crucial role in regulating this sequence of events (14).

At high altitudes, atmospheric pressure is lower. This means that air in a given space expands and, although the oxygen proportion remains the same, the molecules are more dispersed. In the lungs, the number of oxygen molecules in alveoli are reduced, and so there is decreased transfer of oxygen from the alveoli to the bloodstream (15). This is the reason for the fall in SpO_2_ with increase in altitude.

In this review, we only included studies reporting SpO_2_ levels during the period from 24 h after birth to before discharge. Studies reporting SpO_2_ within 24 h of birth were excluded because SpO_2_ levels fluctuate during this period (16). In a study conducted at sea level on healthy neonates, Toth et al. found that the mean post-ductal SpO_2_ was 67% at 2 min after birth, but rose gradually over 14 min to reach 95% (17). Many studies (18–20) on the use of SpO_2_ to screen for congenital heart disease have reported significantly higher false-positive rate when screening is performed within 24 h of birth than when it is performed after 24 h of birth; this too suggests that SpO_2_ levels are unstable during the first 24 h after birth, a period when most of the transition from fetal circulation to neonatal circulation is occurring.

In this study, we excluded preterm infants with gestational age <34 weeks; only full-term or late preterm infants were included. Ravert et al. (21) compared SpO_2_ in term infants and premature infants born at altitudes of 1,371–2,484 meters, and found that preterm infants tend to have higher SpO_2_; however, the authors did not specify the exact gestational age. But, multiple studies (16, 22) on healthy late preterm infants and term infants showed that there was no difference in SpO_2_ between them after birth.

There may be differences between pre-ductal and post-ductal SpO_2_ of neonates at different altitudes. Habib et al. studied healthy neonates born at an altitude of 1,640 meters and found significant difference between pre-ductal and post-ductal SpO_2_ levels in the first 20 min of life (23). A multicenter study from Yunnan, China, also reported significant differences between pre-ductal and post-ductal SpO_2_ levels in healthy neonates during the stable period (i.e., 24 h after birth to before discharge) (5). During screening of neonates for conditions such as congenital heart disease, it is necessary to determine both pre-ductal and post-ductal SpO_2_ (24). We therefore collected the data for both pre-ductal and post-ductal SpO_2_, and built separate prediction equations. Currently, there is no high-quality evidence to indicate whether the larger or smaller integer should be chosen as the lower limit of the reference interval. To minimize the possibility misdiagnosis of hypoxemia in healthy neonates, we chose the smaller integer as the lower limit. Incidentally, the difference between values was not large.

Whenever possible, we preferred to record SpO_2_ values measured in the awake but quiet state. SpO_2_ during sleep can be low due to insufficient ventilation. It is not certain if there is a difference in SpO_2_ between sleeping and awake states at high altitudes. In a study of neonates born at altitudes of 1,371–2,484 meters, Ravert et al. found no significant differences in SpO_2_ values measured at different resting states (21). However, a study on infants aged 1–4 months living at 3,200 meters altitude did find differences in SpO_2_ recorded during awake vs. sleeping states (25). Whether the contradictory results are due to differences in altitude or differences in the ages of the study participants is unclear.

The prediction equation obtained in this systematic review can help guide medical decision making in high altitude areas by sensitively identifying hypoxemia in infants with conditions such as pneumonia, sepsis, and congenital heart disease. In a study conducted in Albuquerque, New Mexico, at an altitude of 1,646 meters, Rao et al. used SpO_2_ of 95% as the threshold to screen for congenital heart disease, and reported a false positive rate of 1.5%, which was much higher than the rate at sea level (26). According to our prediction equation, the lower limit of the reference interval of SpO_2_ at an altitude of 1,646 meters is 92%. However, the sensitivity of using 92% as the threshold needs further study. Previously proposed prediction equations to obtain the SpO_2_ threshold for diagnosis of hypoxemia in children were based on meta-regression analysis (3); we believe that the curve-fitting method we used is more accurate.

Our study has many limitations. In theory, when the data follow a normal distribution, −2SD corresponds to the 2.5th percentile. All studies in this systematic review provided the mean ± standard deviation or standard error. However, we found that many studies had +2SD values (mean values + 2^*^SD) exceeding the theoretical maximum of 100%, suggesting a skewed distribution. Ideally, the results of these studies should have been described using the median and interquartile range, and gave the 2.5th percentile value. We used the curve-fitting method to fit the data, but the accuracy of this approach needs to be verified. Another limitation was that the highest altitude in this study was only 3,100 meters; moreover, for three altitudes, pre-ductal data were not available. The accuracy of the prediction equation for higher altitudes needs to be verified. A third limitation was that factors such as season, ethnicity, POX type, and neonatal resting state during measurement varied between the studies; this may have affected the results. A study from Lhasa, Tibet, found that Tibetan infants had higher SpO_2_ than Han infants at birth and during the first 4 months (27); obviously, altitude is not the only factor affecting SpO_2_ in neonates.

## Conclusions

This is the first systematic review of SpO_2_ in neonates at different altitudes during the stable period. The lower limit of the reference interval of SpO_2_ in healthy neonates tends to decrease as altitude increases. Further research on this topic is needed. Future studies should take into account all factors that could influence SpO_2_ levels, and if the research results are skewed distribution, specific values of the 2.5th percentile in the studies population can be given so as to have a higher quality meta-analysis and systematic review.

## Data Availability Statement

The raw data supporting the conclusions of this article will be made available by the authors, without undue reservation.

## Author Contributions

BW, JZ, and Z-BY designed this work. BW, Y-ZW, and Z-HL extracted the data. BW, JZ, NW, and Z-BY analyzed the data. BW wrote the manuscript. JZ and Z-BY supervised the work. All authors contributed to the article and approved the submitted version.

## Conflict of Interest

The authors declare that the research was conducted in the absence of any commercial or financial relationships that could be construed as a potential conflict of interest.

## Publisher's Note

All claims expressed in this article are solely those of the authors and do not necessarily represent those of their affiliated organizations, or those of the publisher, the editors and the reviewers. Any product that may be evaluated in this article, or claim that may be made by its manufacturer, is not guaranteed or endorsed by the publisher.
