# Supplementary material for: Reference Interval for Pulse Oxygen Saturation in Neonates at Different Altitudes: A Systematic Review
Source: Front Pediatr. 2021 Nov 1;9:771750. doi: 10.3389/fped.2021.771750 (PMC8591307; doi:10.3389/fped.2021.771750)
Supplement: Supplementary file 1 [file Data_Sheet_1.docx]

**PubMed：**

(("Altitude"[Mesh]) OR (Altitudes[Title/Abstract]) OR (elevation[Title/Abstract]) OR (plateau[Title/Abstract]) OR (highland[Title/Abstract]) OR (tableland[Title/Abstract])) AND (("Infant, Newborn"[Mesh]) OR (newborn[Title/Abstract]) OR (newborn*[Title/Abstract]) OR (infant[Title/Abstract]) OR (new born[Title/Abstract]) OR (new borns[Title/Abstract]) OR (newly born[Title/Abstract]) OR (baby*[Title/Abstract]) OR (babies*[Title/Abstract]) OR (premature[Title/Abstract]) OR (prematurity[Title/Abstract]) OR (preterm[Title/Abstract]) OR (pre term[Title/Abstract]) OR (low birth weight[Title/Abstract]) OR (low birthweight[Title/Abstract]) OR (VLBW[Title/Abstract]) OR (LBW[Title/Abstract]) OR (infan*[Title/Abstract]) OR (neonat*[Title/Abstract])) AND (("Oximetry"[Mesh]) OR (Oximetries[Title/Abstract]) OR (Oximetry, Pulse[Title/Abstract]) OR (Oximetries, Pulse[Title/Abstract]) OR (Pulse Oximetries[Title/Abstract]) OR (Pulse Oximetry[Title/Abstract]) OR (Oxygen saturation[Title/Abstract]))
